# Identification of a biomarker panel using a multiplex proximity ligation assay improves accuracy of pancreatic cancer diagnosis

**DOI:** 10.1186/1479-5876-7-105

**Published:** 2009-12-11

**Authors:** Stephanie T Chang, Jacob M Zahn, Joe Horecka, Pamela L Kunz, James M Ford, George A Fisher, Quynh T Le, Daniel T Chang, Hanlee Ji, Albert C Koong

**Affiliations:** 1 Department of Radiation Oncology, Stanford University School of Medicine, Stanford University, Stanford, CA, USA; 2 Stanford Genome Technology Center, Stanford University School of Medicine, Stanford University, Stanford, CA, USA; 3 Department of Biochemistry, Stanford University School of Medicine, Stanford University, Stanford, CA, USA; 4 Department of Genetics, Stanford University School of Medicine, Stanford University, Stanford, CA, USA; 5 Department of Medicine, Division of Medical Oncology, Stanford University School of Medicine, Stanford University, Stanford, CA, USA

## Abstract

**Background:**

Pancreatic cancer continues to prove difficult to clinically diagnose. Multiple simultaneous measurements of plasma biomarkers can increase sensitivity and selectivity of diagnosis. Proximity ligation assay (PLA) is a highly sensitive technique for multiplex detection of biomarkers in plasma with little or no interfering background signal.

**Methods:**

We examined the plasma levels of 21 biomarkers in a clinically defined cohort of 52 locally advanced (Stage II/III) pancreatic ductal adenocarcinoma cases and 43 age-matched controls using a multiplex proximity ligation assay. The optimal biomarker panel for diagnosis was computed using a combination of the PAM algorithm and logistic regression modeling. Biomarkers that were significantly prognostic for survival in combination were determined using univariate and multivariate Cox survival models.

**Results:**

Three markers, CA19-9, OPN and CHI3L1, measured in multiplex were found to have superior sensitivity for pancreatic cancer vs. CA19-9 alone (93% vs. 80%). In addition, we identified two markers, CEA and CA125, that when measured simultaneously have prognostic significance for survival for this clinical stage of pancreatic cancer (*p *< 0.003).

**Conclusions:**

A multiplex panel assaying CA19-9, OPN and CHI3L1 in plasma improves accuracy of pancreatic cancer diagnosis. A panel assaying CEA and CA125 in plasma can predict survival for this clinical cohort of pancreatic cancer patients.

## Background

In 2008, the incidence of pancreatic cancer in the United States was estimated to be more than 38,000, resulting in more than 34,000 deaths per year [1]. Despite being a relatively rare disease, pancreatic cancer is nevertheless the fourth leading cause of cancer death in the United States [2].

Despite the widespread use of aggressive combined modality therapies, the overall 5-year survival for this disease remains less than 5%. Contributing to this high mortality rate is the often late onset of clinical symptoms. The majority of pancreatic cancer is diagnosed when metastases have already occurred (microscopic and gross disease). Since surgical resection is the only therapy associated with long-term survival, there is an urgent need to diagnose patients at an earlier stage of disease when removal of the primary tumor still has curative potential. Issues complicating early diagnosis of pancreatic cancer include the physical location of the pancreas, localized deep within the abdominal cavity, and oftentimes non-specific clinical symptoms such as general abdominal pain, weight loss, and jaundice. Chronic pancreatitis, a common disease encompassing inflammation of the pancreas, can present with identical symptoms. A blood-based diagnostic test has the potential for circumventing these confounding issues, thus enabling earlier detection and increasing the probability of curative surgical treatment.

Currently, carbohydrate antigen 19-9 (CA19-9) is the only plasma marker routinely measured to make clinical decisions pertaining to pancreatic cancer [3]. CA19-9 is most often used to monitor recurrence in resected pancreatic cancer patients as well as to gauge efficacy of chemotherapy and radiotherapy in advanced cases. However, CA19-9 is neither adequately sensitive nor specific enough to make accurate diagnoses of pancreatic cancer based on the results of a serological screening test [4]. CA19-9 is the sialylated Lewis blood group antigen, and as such is not synthesized in approximately 10% of the population [5]. Although a high plasma level of CA19-9 is suggestive of pancreatic cancer in combination with clinical symptoms, imaging studies are usually indicated before any biopsies are undertaken. No other independently measured plasma tumor marker has been shown to exceed CA19-9 in clinical utility.

A panel-based approach simultaneously measuring in multiplex a combination of tumor markers that individually lack optimal sensitivity and specificity has the potential for yielding a diagnostic test with superior characteristics. Previously, we used a multiplex biomarker-measuring technique referred to as proximity ligation assay (PLA) to identify a panel of human plasma biomarkers for pancreatic cancer [6,7]. PLA was initially developed as a technique to improve the sensitivity and specificity of protein detection in a solution-phase, "liquid sandwich ELISA" format [8,9]. As described, this method employs pairs of antibodies coupled to DNA oligonucleotides such that when the antibody pairs bind to the target protein, the local concentration of DNA oligonucleotides increases to allow for enzymatic ligation of the two strands. The resulting amplicons are unique for each specific protein detected and can be measured in a highly quanititative manner by qPCR. Furthermore, PLA can be multiplexed for simultaneous detection of multiple proteins.

PLA has several advantages when compared to current solid-phase approaches. This method of antigen quantification is highly precise; antibody cross-reactivity signal is not observed because of the dual-probe nucleic acid assay design. Also, scalability of the multiplexing is superior to existing methods, since PLA has no upper limit to single-well multiplexing. Bead-based platforms such as Luminex are currently limited to 200-plex assays, although in practice only up to 10 may be used simultaneously due to antibody crossreactivity [10]. Finally, quantification of a PLA is versatile and can be executed on a number of platforms including real-time PCR, mass spectrometry, next-generation sequencing and DNA microarrays. Ultimately, using techniques such as PLA, diagnosis and staging may be improved by detecting a unique pattern of biomarkers that are increased as well as those that are decreased in the plasma of patients displaying clinical symptoms of pancreatic cancer.

In this study, we assembled a cohort of 52 cases of locally advanced, unresectable pancreatic ductal adenocarcinoma (Stage II/III) and 43 healthy, age-matched controls. To date, this dataset represents the largest cohort of pancreatic patients with PLA profiling of putative pancreatic cancer biomarkers. After applying advanced statistical methods to this dataset, we identified a panel of three biomarkers that exceed the diagnostic accuracy of CA19-9 alone. In addition, we identified two biomarkers whose combination are significantly prognostic for survival in advanced, unresectable cancer, as determined by both univariate and multivariate models.

## Materials and methods

### Proximity Ligation Assay

This study probes 21 putative tumor markers for relevance in pancreatic cancer using a proximity ligation assay (PLA). Multiplex PLA was performed on 95 frozen plasma samples as described (3) with the following modifications. Samples were thawed and mixed in a 1:1 ratio with buffer (Olink AB) for undiluted assays or in a 1:50 ratio for diluted assays before incubation for 10 minutes at room temperature. No PDGF-BB spike was added as in previous studies. For probing, we mixed 2 μL of the buffered plasma sample with 2 μL of any one of four probe detection panels validated in the pilot study and incubated the 4 μL mixture for 2 hours at 37°C to allow the probes to bind analytes. Ligation was achieved by incubating 120 μL of reaction mixture with the 4 μL probed samples for 15 minutes at 30°C to dilute and separate any free probes. To stop ligation, 2 μL of uracil-DNA excision mix (Epicentre) was added and incubated for 15 minutes at room temperature.

Preamplification of bar-coded amplicons required mixing 25 μL of ligation reaction mixture with 25 μL of pooled PCR mix (Platinum Taq kit, Invitrogen). After 13 cycles at 95°C for 30 seconds and a 4-minute extension at 60°C, the preamplification products were diluted 10-fold in TE. For each protein assayed, a separate qPCR reaction was required in a 384-well plate with 2 μL of diluted preamplication product sample, 5 μL of iTaq mix (iTaq SYBR Green Supermix with ROX, Bio-Rad), 2 μL qPCR primer mix, and 1 μL water. Protein-specific qPCR detection primers were not dried at the bottom of each well. Real-time qPCR was performed with a sample volume of 10 μL per well for 40 cycles at 95°C for 15 seconds and 60°C for 1 minute. To ensure standardization of values for each biomarker investigated, all 95 samples were simultaneously probed and evaluated on a single 384-well plate with a PBS-BSA blank well.

### Data Processing

Cycle threshold (Ct) values resulting from qPCR were converted into estimated number of starting amplicons, or PLA units, by calculating 10^(-0.301 × Ct+11.439) ^as previously reported (7). After calculating PLA units, data were subsequently transformed into log_2 _space in order to increase normality in the distribution of the data while retaining the magnitude of differences between different tumor markers.

### Human Plasma Samples

This study includes 52 human EDTA blood plasma samples collected between July 2002 and May 2007 from identically staged patients with locally advanced pancreatic ductal adenocarcinoma (Stage II/III) treated at Stanford University Medical Center under an institutional review board-approved protocol. All plasma samples were collected from untreated (*de novo*) patients with biopsy-proven pancreatic adenocarcinomas. Median age at blood collection was 68 years (range 37-84 years). All patients were treated with gemcitabine based chemotherapy and the majority also received radiotherapy. At the end of the study, 41 patients were deceased. As a control group, 43 additional plasma samples were collected from age-matched, healthy volunteers under an IRB-approved protocol. Immediately after acquisition, blood samples were centrifuged and aliquots of plasma stored at -80°C.

### Biomarker Panel Selection and Modeling

All statistical analyses completed in this study were executed using the R statistical computing environment. To select the discrete set of biomarkers used to fit models of pancreatic cancer diagnosis, we used the R distribution of the Prediction Analysis of Microarrays statistical technique, PAMR. Logistic regression models were fit using the generalized linear model function in R.

### Survival Analysis and Modeling

Survival data were fit to a right-censored model using the Survival function in the R statistical computing environment. Univariate and multivariate Cox proportional hazards models were fit onto survival data using the coxph function. Hazard ratios were calculated as the ratios of risk by the increase or decrease of 1 log_2 _PLA unit (2-fold increase or decrease in plasma concentration of a biomarker).

## Results and Discussion

**Figure 1:**
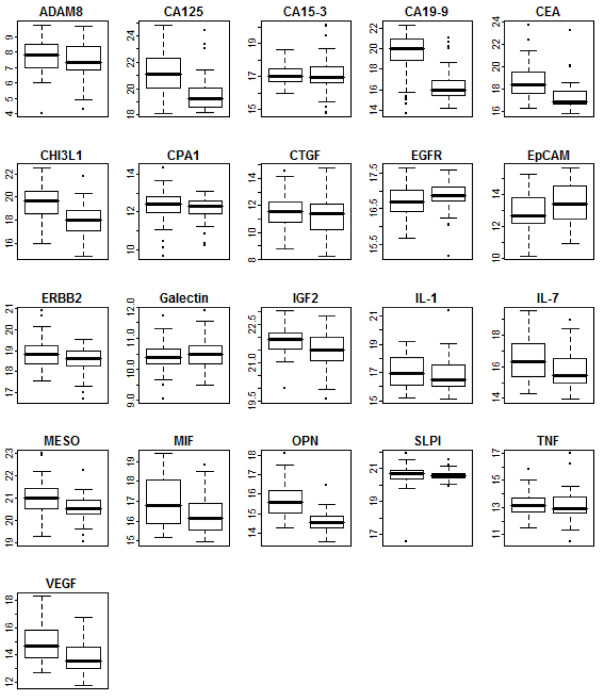
**Plasma levels of 21 tumor markers in pancreatic cancer patients and healthy controls measured by proximity ligation assay**. Each boxplot corresponds to a single tumor marker measured in 95 samples by proximity ligation assay. Pancreatic cancer cases (52) are depicted at left, healthy controls (43) at right. Y-axis corresponds to log_2 _PLA units. Central bars show the median for each cohort, boxes represent the interquartile 50^th ^percentile (IQ50). Whiskers represent 1.5 times the IQ50.

We used a proximity ligation assay (PLA) to measure the levels of 21 tumor markers in the plasma of a cohort of 52 patients with unresectable, advanced pancreatic cancer as well as a cohort of 43 healthy, age-matched volunteers. After calculating log_2 _PLA units for each tumor marker within each sample (Materials and Methods), we initially determined whether any of these tumor markers are significantly elevated or reduced in the plasma of unresectable pancreatic cancer patients compared to healthy controls. To make this comparison, we used the Welch-Satterthwaite modification of Student's *t*-test to determine statistical significance and adjust for unequal variances between cases and controls. Of the 21 tumor markers assayed, we found that 11 were significantly elevated in unresectable pancreatic cancer (*p *< 0.05) (Table 1). One tumor marker, EpCAM, was significant to *p *< 0.04; we would expect approximately 1 tumor marker at this level of significance by random chance given that we assayed 21 tumor markers. We therefore did not consider EpCAM significantly different in cases versus controls. These 11 significant tumor markers were uniformly elevated in pancreatic cancer compared to controls (Figure 1). None of the 21 tumor markers were significantly reduced in pancreatic cancer compared to controls. The tumor marker with the greatest significance of difference was Osteopontin (OPN; *p *< 1.2 × 10^-12^), while the largest magnitude of difference between cases and controls was CA19-9 (approximately 8-fold). Six tumor markers had a greater than 2-fold median elevation in pancreatic cancer compared to controls.

**Table 1:** Proximity ligation assay reveals 11 tumor markers that are significantly elevated in pancreatic cancer cases compared to healthy controls.

Tumor Marker	p * <	Fold Difference^†^	Lower 95% CI	Upper 95% CI
OPN	1.20 × 10^-12^	2.04	14.99	15.38

CA19-9	6.82 × 10^-12^	16.41	17.57	18.55

CHI3L1	8.60 × 10^-8^	3.13	18.42	19.06

CA125	4.86 × 10^-7^	3.54	20.20	20.89

CEA	1.35 × 10^-5^	3	17.70	18.35

VEGF	3.22 × 10^-4^	2.17	14.04	14.65

MESO	0.0014	1.39	20.63	20.92

IGF2	0.0022	1.35	21.45	21.78

IL-7	0.01	1.83	15.88	16.41

MIF	0.01	1.58	16.35	16.88

ERBB2	0.02	1.18	18.57	18.84

EpCam	0.04	0.63	12.85	13.35

EGFR	0.07	0.89	16.58	16.85

IL-1	0.28	1.36	16.72	17.18

ADAM8	0.29	1.35	7.41	7.85

Galectin	0.3	0.94	10.34	10.54

CTGF	0.4	1.12	11.07	11.60

CPA1	0.46	1.07	12.06	12.38

TNF	0.49	1.22	13.03	13.44

SLPI	0.68	1.08	20.41	20.73

CA15-3	0.82	1.02	16.88	17.24

* - *p*-values calculated using Welch-Satterthwaite Student's *t*-test and a two-sided distribution

† - Fold differences calculated comparing cases to controls using log_2 _medians in PLA units

In addition to identifying tumor markers that are significantly elevated in the plasma of pancreatic cancer patients, we investigated whether a panel of tumor markers could diagnose the presence of pancreatic cancer more accurately than the current standard tumor marker for pancreatic cancer, CA19-9. Currently, CA19-9 cannot be used as a practical diagnostic marker because of approximately 80% sensitivity and selectivity rates, as well as an overall 20% error rate. A panel consisting of CA19-9 combined with additional tumor markers could potentially increase the sensitivity and selectivity of tumor marker diagnosis to clinically acceptable levels. To identify an optimal combination of tumor markers that could accurately identify and classify pancreatic cancer cases versus healthy controls on the basis of PLA data, we used an analysis scheme whereby we divided the set of samples randomly into three sets: a discovery set, a modeling set, and a test set. The purpose of the discovery set is to identify the best combination of tumor markers that would most accurately classify cases from controls. To accomplish this discovery step, we used a classification algorithm, PAM (Prediction Analysis of Microarrays) [11]. PAM is a semi-supervised method that uses a shrunken centroid metric to output a sparse number of linear terms that best classifies a dataset. We randomly allocated 50 samples out of 95 to the discovery set. Following the identification of model terms in the discovery step, we next implemented a modeling step to fit coefficients to terms using a logistic regression model of the form:

Where *p*_*i *_is the probability of the *i*th sample being either diagnosed with pancreatic cancer, *b*_*k *_is the coefficient for the *k*th model term, *X*_*k *_is the *k*th model term in the *i*th sample. We randomly allotted 25 samples to the modeling step. We maintained separate discovery and modeling cohorts such that the coefficients of the predictive model would not be subject to optimistic overfitting. Finally, we allotted the remaining 20 samples to a test set to validate the predictive quality of the logistic regression model. We validated using a test set rather than a crossvalidation approach because crossvalidation in general is overly optimistic, and we hoped to identify a panel of biomarkers that could be implemented clinically. Because the test set sample size is small, only 20 samples, to address the potential for a test set to be either overly optimistic or pessimistic due to random selection, and gauge the robustness of the data, we repeated the discovery, modeling, and test set validation steps 10 times, each time randomly assigning samples, recalculating model terms via PAM, refitting model coefficients, and independently testing the validity of the model. At no time during our analysis of the data was there any overlap in training and test sets for any of the 10 independent test runs, nor was there any overlap in analysis between any of the test runs. There existed the potential that several models with differing model terms could have been outputted from test run to test run. For each test run, we tabulated model terms, sensitivity, selectivity and error frequency, and compared the multi-marker panel model to results for a model incorporating CA19-9 only.

**Figure 2:**
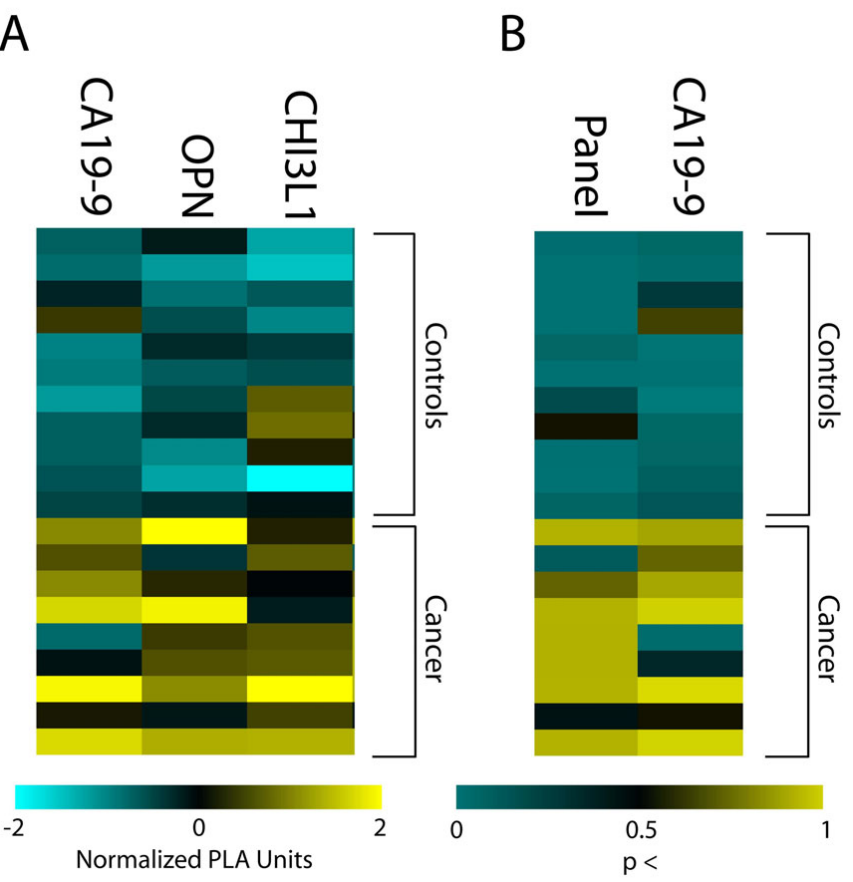
**A tumor marker panel consisting of CA19-9, OPN, and CHI3L1 predicts the presence of pancreatic cancer more accurately than CA19-9 alone**. **(A) **Each row corresponds to 1 of 20 randomly assigned pancreatic cancer cases or healthy controls in the test set. Each column represents a tumor marker. Cells depict normalized log_2 _PLA units. (**B) **Rows are as A. Columns represent either a three-marker panel consisting of CA19-9, OPN, and CHI3L1, or CA19-9 alone. Cells depict the model-outputted probability that a given sample is either pancreatic cancer or healthy control, with a cutoff of *p *> 0.5 to be considered pancreatic cancer.

After completing this analysis, we found that in 10 out of 10 independent test runs, PAM identified a panel of the same three tumor markers, CA19-9, OPN, and CHI3L1, as the optimal terms to classify pancreatic cancer from healthy controls. When comparing sensitivity and selectivity of the tumor marker panel to CA19-9 alone, we found that the tumor marker panel showed a significant increase in sensitivity (0.93 vs. 0.81) (Table 2). Selectivity was approximately similar between the panel and CA19-9 alone. We also calculated average positive predictive value (0.83 vs. 0.80) and average negative predictive value (0.93 vs. 0.79). Finally, overall errors in prediction made by the three tumor marker panel were approximately 60% in frequency compared to CA19-9 alone. We conclude that a panel consisting of CA19-9, OPN, and CHI3L1 is superior for pancreatic cancer diagnosis compared to CA19-9 alone (Figure 2).

**Table 2:** Analysis of diagnostic sensitivity, selectivity and error for a panel consisting of CA19-9, OPN and CHI3L1 compared to CA19-9 alone.

Test Run*	Panel Sensitivity^†^	Panel Selectivity^‡^	Panel Error^§^	CA19-9 Sensitivity^||^	CA19-9 Selectivity**	CA19-9 Error^††^
1	0.92 (0.65 - 0.99)	0.88 (0.53 - 0.98)	0.10	0.92 (0.65 - 0.99)	0.88 (0.53 - 0.98)	0.10

2	1.00 (0.65 - 1.0)	0.69 (0.42 - 0.87)	0.10	0.33 (0.14 - 0.61)	0.75 (0.41 - 0.93)	0.50

3	1.00 (0.65 - 1.0)	0.69 (0.42 - 0.87)	0.10	1.00 (0.65 - 1.0)	0.62 (0.36 - 0.82)	0.25

4	1.00 (0.76 - 1.0)	0.88 (0.53 - 0.98)	0.05	0.92 (0.65 - 0.99)	1.00 (0.68 - 1.0)	0.05

5	1.00 (0.68 - 1.0)	0.92 (0.65 - 0.99)	0.15	1.00 (0.68 - 1.0)	0.83 (0.55 - 0.95)	0.10

6	0.89 (0.57 - 0.98)	0.82 (0.52 - 0.95)	0.05	0.89 (0.57 - 0.98)	0.45 (0.21 - 0.72)	0.35

7	0.75 (0.47 - 0.91)	0.75 (0.41 - 0.93)	0.05	0.67 (0.39 - 0.86)	0.75 (0.41 - 0.93)	0.30

8	1.00 (0.72 - 1.0)	0.80 (0.49 - 0.94)	0.10	0.90 (0.60 - 0.98)	0.80 (0.49 - 0.94)	0.15

9	1.00 (0.77 - 1.0)	0.71 (0.36 - 0.92)	0.10	0.69 (0.42 - 0.87)	1.00 (0.65 - 1.0)	0.20

10	0.78 (0.45 - 0.94)	1.00 (0.74 - 1.0)	0.10	0.67 (0.35 - 0.88)	0.91 (0.62 - 0.98)	0.20

**Average**	**0.93**	**0.81**	**0.13**	**0.80**	**0.80**	**0.22**

*- One complete run of analysis, including random sample division into training, modeling, and test sets

† - Sensitivity of logistic regression model prediction with CA19-9, OPN, and CHI3L1 as model terms. Parenthetical values represent the 95% CI.

‡ - Selectivity of logistic regression model prediction with CA19-9, OPN, and CHI3L1 as model terms. Parenthetical values represent the 95% CI.

§- Frequency of combined false negative and false positive calls in 20 test samples using a logistic regression model with CA19-9, OPN, and CHI3L1 as model terms

|| - Sensitivity of logistic regression model prediction with CA19-9 alone as a model term. Parenthetical values represent the 95% CI.

**- Selectivity of logistic regression model prediction with CA19-9 alone as a model term. Parenthetical values represent the 95% CI.

†† - Frequency of combined false negative and false positive calls in 20 test samples using a logistic regression model with CA19-9 alone as a model term

**Figure 3:**
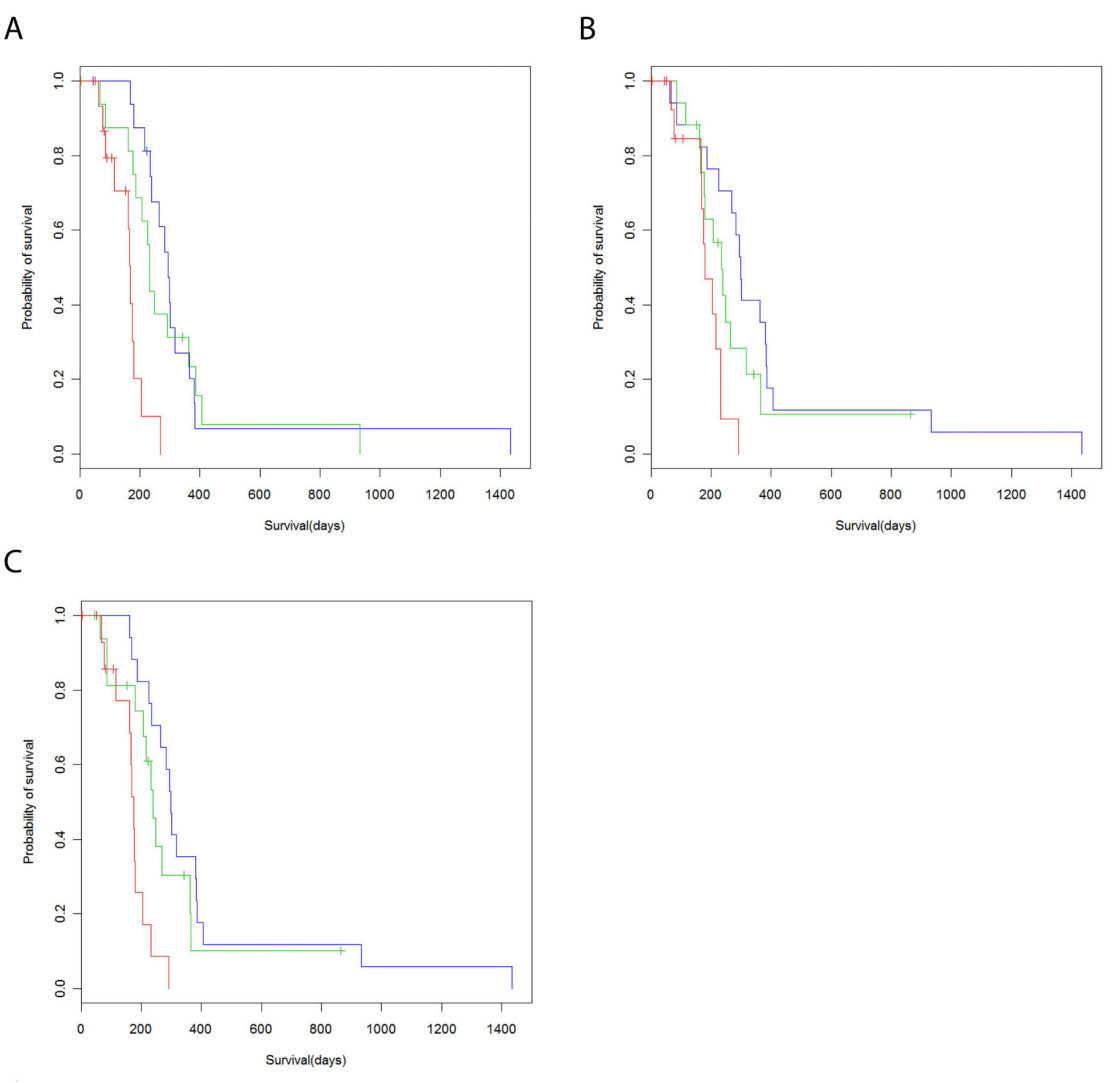
**CEA and CA125 are significantly prognostic for advanced, unresectable pancreatic cancer**. **(A) **Kaplan-Meier plot depicting survival of 52 cases of advanced, unresectable pancreatic cancer. Cohort divided into tertiles by CEA plasma levels measured by proximity ligation assay. Red line denotes highest 33% by CEA plasma level, green line medial 33%, and blue line lowest 33%. Tick marks represent right censored data. **(B) **Cohort divided into tertiles by CA125 plasma levels measured by proximity ligation assay. Otherwise as A. **(C) **Cohort divided into tertiles by combined, rank-ordered levels of CEA and CA125 as measured in plasma by PLA. Otherwise as A.

Beyond diagnosing pancreatic cancer, we were interested in identifying tumor markers that are prognostic for post-draw survival in advanced, unresectable pancreatic cancer. To accomplish this, we fit the survival of the 52 pancreatic cancer cases to a Cox proportional hazards model of the form:

where *h(t) *is the hazard function at time *t*, *h*_0_*(t) *is the hazard function when the value of all independent variables is zero, *b*_*k *_is the coefficient for the *k*th model term, and *X*_*k *_is the *k*th model term. We fit both a univariate model considering only the plasma level of tumor markers as measured by the PLA, as well as a multivariate model considering tumor marker level, gender, and whether the patient was treated by radiotherapy (Table 3). Under both models, only two tumor markers were significantly prognostic: CEA and CA-125. Of the two, CEA is the most prognostic. After observing this result, we also considered that a combined multivariate Cox model using CEA, CA125, gender, and radiotherapy would be more prognostic than a multivariate model containing either tumor marker alone. A combined model did prove to be superior (log likelihood *p *< 0.003). We also considered a multivariate model involving radiotherapy, ECOG performance score, and serum albumin in combination with each of 21 biomarkers. As in previous models, only CA125 and CEA were shown to be significantly prognostic (*p *< 0.05; Table 4). Following this, we divided the 52 cases into tertiles by CEA, CA125, or both (Figure 3). The median patient in the lower third of CEA and CA125 level will survive approximately 4 months longer than the median patient in the upper third. We therefore conclude that a panel of tumor markers consisting of CEA and CA125 can prognostically stratify cases of unresectable pancreatic cancer.

**Table 3:** Univariate and multivariate Cox proportional hazard models fit on 21 tumor markers.

Tumor Marker	p* <	HR^†^	*p*^‡ ^<	HR^§^
CEA	0.00019	1.54 (1.23 - 1.93)	0.0007	1.55 (1.21 - 2.05)

CA125	0.0014	1.45 (1.16 - 1.83)	0.0025	1.43 (1.14 - 1.80)

EGFR	0.089	2.17 (0.89 - 5.30)	0.12	2.16 (0.81 - 5.75)

CPA1	0.13	1.33 (0.92 - 1.94)	0.023	1.54 (1.06 - 2.24)

ERBB2	0.24	1.31 (0.84 - 2.03)	0.0023	1.84 (1.23 - 2.76)

ADAM8	0.26	1.20 (0.87 - 1.66)	0.51	1.12 (0.80 - 1.58)

CA15-3	0.27	1.33 (0.80 - 2.20)	0.3	1.33 (0.77 - 2.30)

SLPI	0.27	1.32 (0.80 - 2.15)	0.005	1.86 (1.21 - 2.87)

MIF	0.31	0.88 (0.68 - 1.13)	0.36	0.88 (0.67 - 1.16)

Galectin	0.34	1.33 (0.74 - 2.41)	0.36	1.35 (0.72 - 2.55)

IGF2	0.37	1.25 (0.77 - 2.02)	0.042	1.63 (1.02 - 2.62)

MESO	0.42	1.18 (0.79 - 1.74)	0.062	1.45 (0.98 - 2.16)

CTGF	0.45	1.09 (0.88 - 1.34)	0.98	1.00 (0.78 - 1.27)

TNF	0.47	1.13 (0.82 - 1.56)	0.17	1.25 (0.91- 1.71)

VEGF	0.58	0.94 (0.74 - 1.19)	0.65	0.94 (0.73- 1.22)

IL-7	0.58	0.95 (0.78 - 1.15)	0.52	0.93 (0.75 - 1.16)

EpCAM	0.61	1.07 (0.83 - 1.37)	0.35	1.14 (0.86 - 1.52)

CA19-9	0.67	1.04 (0.88 - 1.23)	0.86	0.98 (0.82 - 1.18)

OPN	0.68	1.10 (0.71 - 1.69)	0.58	0.87 (0.54 - 1.41)

IL-1	0.85	0.97 (0.74 - 1.28)	0.42	0.88 (0.65 - 1.19)

CHI3L1	0.94	0.99 (0.78 - 1.27)	0.91	0.99 (0.76 - 1.28)

*- *p*-value derived from a univariate Cox proportional hazards model accounting for the effect of tumor marker only on prognosis

† - Hazard ratio derived from univariate Cox proportional hazards model. Parenthetical values denote 95% confidence interval.

‡ - *p*-value derived from a multivariate Cox proportional hazards model accounting for tumor marker, sex, and therapy on prognosis

§- Hazard ratio derived from multivariate Cox proportional hazards model. Parenthetical values denote 95% confidence interval.

**Table 4:** Multivariate Cox proportional hazards on radiotherapy, ECOG performance score, serum albumin and 21 tumor markers

Tumor Marker	p* <	HR^†^
CA125	0.033	1.37 (1.02 - 1.99)

CEA	0.037	1.43 (1.03 - 1.82)

CPA1	0.082	1.43 (0.60 - 4.33)

Adam8	0.14	1.29 (0.96 - 2.14)

Erbb2	0.17	1.42 (0.86 - 2.34)

SLPI	0.24	1.38 (0.92 - 1.81)

MESO	0.28	1.31 (0.56 - 1.81)

EGFR	0.34	1.61 (0.81 - 2.34)

VEGF	0.43	1.13 (0.75 - 1.35)

TNF	0.48	1.14 (0.61 - 2.71)

IL-7	0.54	1.07 (0.66 - 1.88)

CTGF	0.55	1.08 (0.80 - 2.14)

CA19-9	0.64	0.96 (0.84 - 1.38)

EpCam	0.51	1.12 (0.80 - 1.62)

Galectin	0.51	1.28 (0.83 - 1.54)

MIF	0.95	0.99 (0.85 - 1.36)

OPN	0.68	1.12 (0.80 - 1.57)

CHI3L1	0.8	0.96 (0.82 - 1.13)

IGF2	0.68	1.12 (0.64 - 1.97)

CA15-3	0.98	1.01 (0.80 - 1.57)

IL-1	0.5	1.12 (0.69 - 1.33)

*- *p*-value derived from a univariate Cox proportional hazards model accounting for the effect of tumor marker only on prognosis

† - Hazard ratio derived from univariate Cox proportional hazards model. Parenthetical values denote 95% confidence interval.

## Conclusions

This study of 52 cases and 43 controls is the largest sample set of pancreatic cancer patients in which PLA was used for multiplexed detection of secreted proteins. All patients were identically staged and were determined to have locally advanced pancreatic cancer (Stage II/III). Furthermore, all plasma samples were obtained prior to initiating any therapy. From this carefully defined clinical population, we conclude that a 3-member plasma biomarker panel consisting of CA19-9, osteopontin (OPN), and chitinase 3-like 1 (CHI3L1) resulted in improved diagnostic accuracy compared to CA19-9 alone for locally advanced, unresectable tumors.

CA19-9 is the most widely used biomarker in pancreatic cancer, but its use is primarily limited to monitoring responses to cancer therapy and recurrence of resected tumors and plays only a minor role in diagnosis. CA19-9 can be falsely elevated in patients with benign pancreatico-biliary conditions such as cholestasis and pancreatitis. Furthermore, this Lewis blood group antigen is not expressed in up to 10% of the population [12]. Although the combination of CA19-9, OPN, and CHI3L1 improves the diagnostic accuracy compared to CA19-9 alone, our study was limited to patients with locally advanced pancreatic cancer. Although extrapolation of these data to an asymptomatic population as a potential screening tool would not be appropriate, our results suggest that the use of biomarker panels for the initial diagnosis of pancreatic cancer is promising. Increased or decreased levels of specific proteins in the blood may indicate important information regarding the underlying biology of pancreatic cancer.

Other investigators have reported that CHI3L1 (also known as YKL-40) is an important biomarker for breast and ovarian cancer [13-17]. In solid tumors, this protein has been shown to be important in the regulation of extracellular matrix remodeling, suggesting a role in invasion and metastases [18]. Interestingly, CHI3L1/YKL-40 was found in a prospective Danish population study to be predictive of ultimately developing gastrointestinal cancer. Furthermore, elevation of this biomarker also predicted decreased survival after diagnosis [19].

Osteopontin is an important biomarker in head and neck cancer [20,21] as well as lung cancer [22], and has been shown to be in involved in angiogenesis by acting through the PI3K/Akt pathway to enhance the expression of VEGF [23]. In pancreatic cancer, Koopmann et al demonstrated that serum OPN levels were significantly elevated in patients with pancreatic adenocarcinoma prior to surgical resection compared to healthy controls. Based upon serum ELISA, these investigators reported a sensitivity of 80% and a specificity of 97% [24]. OPN is a secreted protein responsible for stimulating various signaling pathways, including those promoting survival and metastases under hypoxia [25]. This protein also functions as a chemotactic factor for macrophages, dendritic cells, and T cells. Depending upon the context, OPN has been shown to have both pro- and anti-inflammatory functions [26].

We previously reported in a smaller study of 20 patients that an 11 biomarker panel (CA19-9, CHI3L1, OPN, CA-125, ERBB2, ADAM8, SLPI, IGF-2, VEGF, CTGF) resulted in increased diagnostic accuracy compared to CA 19-9 alone [7]. However, in the current study, only CA19-9, CHI3L1, and OPN retained significance in improving diagnostic accuracy. In the previous study, although Prediction Analysis of Microarrays was used to calculate a panel, no modeling steps were carried out to optimize the predictive value of a biomarker panel. Furthermore, k-fold crossvalidation rather than an independent test set was used to validate the panel hypothesis; k-fold crossvalidation has the disadvantage of being statistically optimistic. The present study also has the advantage of increased size and statistical resolution, considering greater than twice as many cases compared to the previous study. We postulate that these factors account for the update in findings between these two studies. In addition to our studies using PLA to find multiplex panels for the diagnosis of pancreatic cancer, recent work using the LabMAP technology platform identified a panel of cytokines in plasma that can detect pancreatic cancer with higher specificity than CA19-9 measured alone using traditional ELISA methods [27].

In this study, we found that a combination of CEA and CA125 has superior prognostic value for locally advanced pancreatic cancer in two survival models. CEA has been previously shown to have some value for predicting survival in pancreatic cancer [28], and although CEA is usually measured in the context of diagnosing colorectal cancer, this marker has also been shown to be elevated in approximately half of all pancreatic cancer cases [29]. CA125 is a commonly measured marker of ovarian cancer used in the diagnosis and treatment of that neoplasm [30,31]. To date, no studies have implicated CA125 for utility in pancreatic cancer prognosis.

It is unlikely that a single biomarker will result in 100% sensitivity and 100% specificity for pancreatic cancer. However, continued progress in biomarker discovery efforts may one day yield a panel of biomarkers that will approach the sensitivity and specificty required for screening large populations with a blood test. The greatest utility of such a test would be to identify those individuals with precancerous lesions such as pancreatic intrepithelial neoplasia (PanIN) or intraductal papillary mucinous tumor (IPMT). Because most of these lesions are microscopic and noninvasive, it is unlikely that a blood test will have sufficient sensitivity to detect these lesions. Biomarker profiling of pancreatic juice obtained endoscopically is another strategy that some investigators are using to overcome this limitation. Although PLA has not yet been used to characterize biomarker profiles in pancreatic juice, in theory, this technology could be applied to this fluid which should further increase diagnostic accuracy.

## Competing interests

The authors declare that they have no competing interests.

## Authors' contributions

STC, JMZ, and JH carried out Proximity Ligation Assay experiments. STC and JMZ executed data analysis and statistical data modeling. PLK, JMF, GAF, QTL, DTC, HJ, and ACK conceived of experiments and data analyses. STC, PLK, JMF, GAF, QTL, DTC, HJ, and ACK collected specimens and coordinated clinical data. All authors read and approved this manuscript.
